# Revision 1 Size and position of the healthy meniscus, and its Correlation with sex, height, weight, and bone area- a cross-sectional study

**DOI:** 10.1186/1471-2474-12-248

**Published:** 2011-10-28

**Authors:** Katja Bloecker, Martin Englund, Wolfgang Wirth, Martin Hudelmaier, Rainer Burgkart, Richard B Frobell, Felix Eckstein

**Affiliations:** 1 Institute of Anatomy & Musculoskeletal Research, Paracelsus Medical University (PMU) Salzburg, Austria; 2 Department of Orthopedics, Clinical Sciences Lund, Lund University, Lund, Sweden; 3 Clinical Epidemiology Research & Training Unit, Boston University School of Medicine, Boston, MA, USA; 4 Chondrometrics GmbH, Ainring, Germany; 5 Clinic for Orthopaedics and Traumatology, Technical University Munich, Germany; 6 Department of Orthopedics, Clinical Sciences Lund, Lund University, Lund, Sweden

## Abstract

**Background:**

Meniscus extrusion or hypertrophy may occur in knee osteoarthritis (OA). However, currently no data are available on the position and size of the meniscus in asymptomatic men and women with normal meniscus integrity.

**Methods:**

Three-dimensional coronal DESSwe MRIs were used to segment and quantitatively measure the size and position of the medial and lateral menisci, and their correlation with sex, height, weight, and tibial plateau area. 102 knees (40 male and 62 female) were drawn from the Osteoarthritis Initiative "non-exposed" reference cohort, including subjects without symptoms, radiographic signs, or risk factors for knee OA. Knees with MRI signs of meniscus lesions were excluded.

**Results:**

The tibial plateau area was significantly larger (p < 0.001) in male knees than in female ones (+23% medially; +28% laterally), as was total meniscus surface area (p < 0.001, +20% medially; +26% laterally). Ipsi-compartimental tibial plateau area was more strongly correlated with total meniscus surface area in men (r = .72 medially; r = .62 laterally) and women (r = .67; r = .75) than contra-compartimental or total tibial plateau area, body height or weight. The ratio of meniscus versus tibial plateau area was similar between men and women (p = 0.22 medially; p = 0.72 laterally). Tibial coverage by the meniscus was similar between men and women (50% medially; 58% laterally), but "physiological" medial meniscal extrusion was greater in women (1.83 ± 1.06mm) than in men (1.24mm ± 1.18mm; p = 0.011).

**Conclusions:**

These data suggest that meniscus surface area strongly scales with (ipsilateral) tibial plateau area across both sexes, and that tibial coverage by the meniscus is similar between men and women.

## Background

Osteoarthritis (OA) is the most common type of arthritis, predominantly affecting the knees, hips, and hands [1]. Knee OA is more frequent [1-3] and shows faster progression in women than in men [1]. The reason why women develop knee OA more frequently than men is currently not understood.

The menisci transmit forces in the knee during dynamic and static conditions [4,5] and have been reported to transfer 30-55% of the knee loads in a standing position [6]. By distributing loads and reducing the knee joint contact stress, the menisci keep the forces encountered by the cartilage and subchondral bone tissue in reasonable limits [4,5,7] and thus protect the joint from developing OA [8]. It is well known that meniscectomy increases peak loads in the knee [6,9] and dramatically increases the incidence of knee OA [10-16].

Recently, a magnetic resonance image (MRI)-based method for quantitative analysis of meniscus size, shape and position has been presented [17] and shows satisfactory intra-observer [17] and inter-observer precision in vivo [18]. Given the higher prevalence of knee OA in women and the important role of the meniscus in knee joint biomechanics and OA development, we explored whether meniscus size, shape or position differ between asymptomatic men and women who do not have clinical knee OA. Since the meniscus is known to undergo morphological changes in OA [14,17], this hypothesis needs to be tested under physiological conditions, prior to the onset of disease. For this reason, the morphology of the meniscus was studied in asymptomatic and radiographically normal men and women without MRI signs of meniscus lesions.

Since men are obviously larger (and heavier) than women, a comparison of morphometric parameters of the meniscus between both sexes is not straight forward. It was therefore examined to what extent the size of the normal meniscus correlates with the body height, weight, and tibial plateau area, in men and women. This can provide clues, as to which of these anthropometric variables are best suited to account for size differences between subjects and both sexes, and whether a relative measure of meniscus size can be derived that can be objectively compared between men and women.

Studying this relationship also is valuable for the design of patient-specific meniscus transplants, and for elucidating the role of the meniscus in synovial joint pathology. Recent studies [17,19] suggested that the meniscus hypertrophies in subjects with knee OA compared with healthy ones. Given the relatively large variability of size-dependent morphometric parameters in the population, however, the power of such comparative analysis remains low, as long as meniscus size is not related to the individual. Also, such comparative studies are less powerful if men and women (with and without knee OA) need to be studied separately, rather than together. A relevant question in this context therefore is, to what extent measures of meniscus size differ by sex, body size, and age, and whether a "relative" (i.e. normalized) measure can be obtained that displays less variability between individuals than absolute measures of meniscus size. Similar considerations apply to quantitative measures of meniscal maceration (i.e. loss of substance).

This study therefore addressed the following questions: 1) Do absolute (and relative) measures of the size and position of the medial or lateral meniscus differ between asymptomatic men and women without radiographic knee OA? 2) What is the correlation between measures of meniscus size with body height, body weight, tibial plateau area, and age? 3) Can a relative (normalized) measure of meniscus size be proposed that displays less variability between subjects (and sexes) than absolute measures, and that can be used in quantitative studies examining meniscus hypertrophy (or substance loss) in knee OA?

## Methods

### Study participants

Three Tesla MR images (public-use data set 0.F.1; [20,21]) were obtained from the 122 participants (47 men; 75 women) of the „non-exposed" reference cohort of the Osteoarthritis Initiative (OAI: http://oai.epi-ucsf.org/datarelease/). Inclusion criteria were: no history of pain, aching or stiffness in either knee in the past year, no radiographic findings of femorotibial OA [22], no risk factors for the onset of knee OA including obesity, knee surgery, history of knee injury, family history of total knee replacement, OA in the hands, or repetitive knee bending in work or other daily activities. The MR images of three women were missing, and those of two other women were of insufficient quality, so that MRI data from 117 knees was available. Because asymptomatic participants without radiographic knee OA are known to commonly display meniscus damage [23], knees with meniscus lesions on MRI were excluded. To this end, one clinical investigator (M.E.) read all knee MR images for the presence of meniscus tears or maceration/destruction semi-quantitatively using the BLOKS scoring system [24] on the intermediately-weighted sagittal and coronal turbo spin-echo (IWTSE) sequences. Knees showing any signs of meniscus lesions (n = 15) were excluded from the study. Consequently, 62 women (mean age 54 [range 46-69] years; body height 1.64 ± 0.06 m; weight 62.3 ± 8.4kg; BMI 23.2 ± 2.7kg/m^2^) and 40 men (57 [45-79] years); body height 1.74 ± 0.07 m; weight 79.2 ± 8.2 kg; BMI 26.1 ± 2.9kg/m^2^) were studied. All patients gave informed consent to participate in this study. Additionally the study was approved by the institutional review board for the University of California, San Francisco (UCSF) and its affiliates (approval nr. H5254-20499-09).

**Figure 1:**
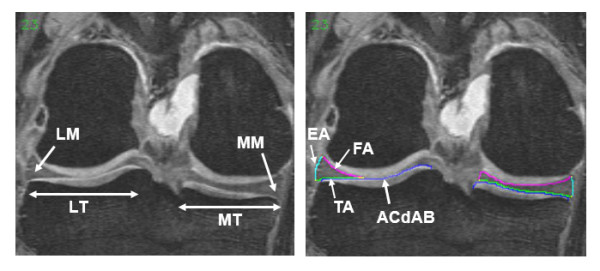
**Coronal MR images of the right knee without segmentation (left) and with segmentation (right) of the medial (MM) and lateral meniscus (LM)**. The segmented surface of the tibial plateau area (ACdAB) of the medial (MT) and lateral tibia (LT) is shown in blue. The segmented surfaces of the meniscus are shown in green (tibial area = TA), magenta (femoral area = FA), and turquoise (external area = EA).

Coronal multi-planar reconstructions of the sagittal double echo steady state sequence with water excitation (MPR DESSwe [25-29]) of the right knees were used (Figure 1; repetition time = 16.3ms, echo time = 4.7ms, flip angle = 25°, reconstructed slice thickness = 1.5mm, in-plane resolution 0.37mm × 0.7mm, interpolated to 0.37mm × 0.37mm). Manual segmentation of the the medial and lateral tibial plateau area (area of cartilage surface, including denuded areas of subchondral bone = ACdAB), the tibial area (TA), the femoral area (FA), and the external area (EA) of the medial (MM) and lateral meniscus (LM) was performed by a single operator (K.B.) using specialized image analysis software (Chondrometrics GmbH, Ainring, Germany) [17,30] (Figure 1). In the first 20 cases, coronal intermediately weighted turbo spin echo (IW TSE) images, commonly used to evaluate meniscus pathology [31-33] were displayed in parallel to assist the meniscal segmentation. For an explanation of the nomenclature used, please see [17,30]. Segmentation started anteriorly, ended posteriorly, and was restricted to slices, in which both the tibial cartilage and the meniscus could be reliably identified. Internally, the borders of the menisci were defined by the internal margin of the cartilage surface of MT and LT, because these are continuous with the transverse and menisco-femoral ligament and because no intrinsic anatomical demarcation could be used to separate these structures. The segmentations were quality controlled by a second reader (F.E.) and adjustments were made by consensus.

**Figure 2:**
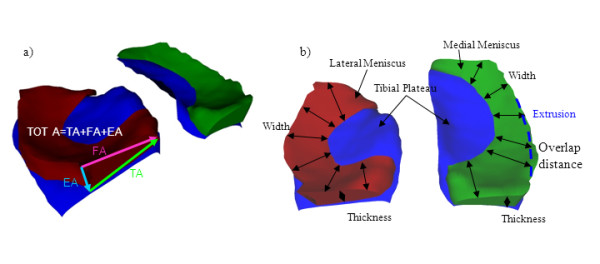
**3D reconstruction of the medial meniscus (green) and lateral meniscus (red); a) meniscal areas are marked (see Figure 1)**. The total surface area of the meniscus (TOT A) = TA+FA+EA; b) the width and thickness of the meniscus are shown with arrows. The extrusion is measured by the area/distance between the blue intersected line (external edge of the tibial plateau) and the external edge of the meniscus TA. The coverage of the tibial plateau area is determined by the area/distance (i.e. overlap distance) between the blue intersected line and the internal edge of TA.

The size of ACdAB, TA, FA and EA, and the sum of the three meniscal surfaces (TOT A) were computed after 3D triangulation [17,34] (Figure 2). The coverage of ACdAB by TA was determined in absolute (mm^2^) and relative measures (%) [17] (Figure 2). Additionally, the volume (V), mean thickness (Th.Me), maximal thickness (Th.Max [also termed meniscal height by other authors [35]), mean (Wid.Me) and maximal meniscal width (Wid.Max) were determined, as described previously [17]. Meniscal position (relative to the tibial plateau area) was determined as the absolute and relative area of the TA not covering the ACdAB (TA.Uncov). Further, we computed the mean (and maximal distance between the external margin of ACdAB and TA [17]. These measures were termed mean (Ex.Me) and maximal "physiological" external meniscal extrusion (Ex.Max). This terminology is not meant to refer to a pathological condition but to describe the position of the meniscus in asymptomatic volunteers, e.g. under "physiological" conditions. The position of the "internal" margin of the meniscus was determined as the mean (OvD.Me) and maximum overlap distance (OvD.Max), i.e. the distance between the external margin of ACdAB and the intersection of TA and FA.

Mean values, the standard deviation (SD), and the coefficient of variation (CV% = SD/mean) were determined for all measures in men and women, and sex differences were assessed using a two-tailed, unpaired t-test. Pearson correlation coefficients between measures of meniscus size and age, body height, body weight, and bone area (ipsi-compartimental tibial ACdAB, contra-compartimental ACdAB, and total ACdAB) were determined by linear regression.

## Results

**Figure 3:**
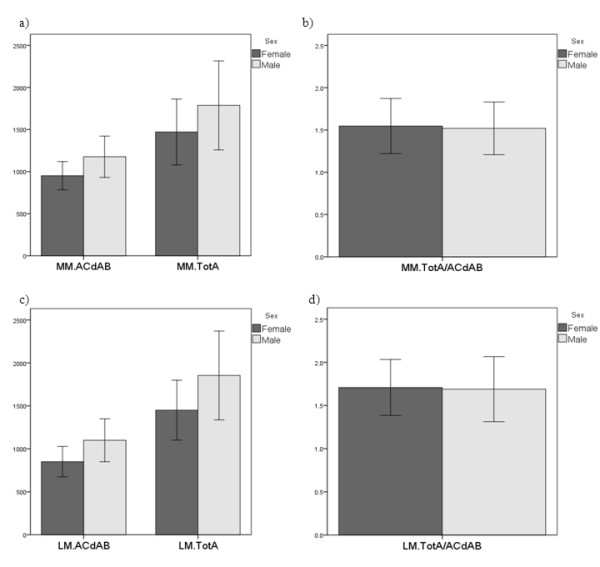
**Bar graphs showing the size of the tibial plateau area (ACdAB), the total surface area of the meniscus (TOT A) and the ratio of TOT A/ACdAB in men and women**. The error bars show ± 2 standard deviations: a+b) Medial tibial plateau (MT) and medial meniscus (MM); c+d) Lateral tibial plateau (LT) and lateral meniscus (LM).

The medial tibial plateau area was 23% larger in men than in women (Table 1), and the lateral plateau was 28% larger (Table 2; Figure 3). The medial meniscus displayed a 30% greater volume, a 10%/8% greater mean/maximal thickness, and a 20% larger total surface area (TOT A) in men than in women (Table 1; Figure 3). In the lateral menisci, the volume was 30% greater, the mean/maximal thickness 6%/7% greater, and the TOT A 27% larger in men than in women (Table 2; Figure 3).

**Table 1:** Size and position of the medial meniscus in men and women

	Men Mean	SD	Women Mean	SD	Diff (%)	t-test
**Tibial Plateau and Meniscus size**						
ACdAB	1169	122	952	85.7	22.7	<0.001
V	2407	520	1853	321	29.9	<0.001
TA	620	93.0	518	65.9	19.6	<0.001
FA	714	117	597	73.7	19.8	<0.001
EA	440	75.4	365	56.2	20.5	<0.001
TOT A	1774	276	1480	178	19.9	<0.001
TOT A/ACdAB	1.52	0.161	1.56	0.143	-2.45	0.221
Th.Me	2.80	0.292	2.55	0.245	10.1	<0.001
Th.Max	7.71	1.15	7.15	0.893	7.84	0.008
Wid.Me	9.93	1.06	9.11	0.875	8.99	<0.001
Wid.Max	18.8	2.08	16.9	1.61	11.1	<0.001
**Meniscus position**						
TA.Uncovp	10.0	4.42	12.2	4.05	-18.2	0.012
mEx.Me	1.24	1.18	1.83	1.06	-32.4	0.011
mEx.Max	8.05	1.57	7.55	1.75	6.65	0.149
ACdAB.Covp	49.6	5.72	49.8	5.37	-0.36	0.874
OvD.Me	-12.0	1.31	-10.7	1.28	12.6	<0.001
OvD.Max	-4.66	1.44	-4.04	1.15	15.2	0.021

SD: standard deviation; Diff(%): difference in percent; t-Test: unpaired t-test; ACdAB: area of cartilage surface, including denuded areas of subchondral bone; V: volume; TA: tibial area; FA: femoral area; EA: external area; TOT A: sum of all three surface areas of the meniscus; Th.Me: mean thickness of the meniscus; Th.Max: maximal thickness of the meniscus; Wid.Me: mean width of the meniscus; Wid.max: maximal width of the meniscus; TA.uncovp: tibial area of the meniscus not covering the tibial plateau in percent; Ex.Me: mean external extrusion; Ex.Max: maximal external extrusion; ACdAB.Covp: area of cartilage surface covered with meniscus in percent; OvD.Me: mean overlap distance; OvD.Max: maximal overlap distance. Note that a positive value for meniscal extrusion indicates an "external" position relative to the external border of the tibial plateau, whereas a negative value indicates an "internal" position relative to the external border. A more negative value for the overlap distance indicates a more internal position of the inner margin of the meniscus.

**Table 2:** Size and position of the lateral meniscus in men and women

	Men Mean	SD	Women Mean	SD	Diff (%)	t-test
**Tibial Plateau and Meniscus size**						
AcdAB	1101	121	864	87.2	27.5	<0.001
V	2441	487	1824	296	33.9	<0.001
TA	651	86.6	503	60.7	29.5	<0.001
FA	746	116	581	76.8	28.4	<0.001
EA	451	69.7	379	51.4	18.8	<0.001
TOT A	1848	260	1463	174	26.3	<0.001
TOT A/AcdAB	1.68	0.201	1.70	0.134	-0.72	0.720
Th.Me	2.67	0.260	2.51	0.237	6.23	0.003
Th.Max	7.23	0.980	6.75	0.976	7.11	0.019
Wid.Me	10.1	1.28	8.60	0.780	16.9	<0.001
Wid.Max	14.2	2.14	12.5	1.55	13.0	<0.001
**Meniscus position**						
TA.Uncovp	6.36	3.68	5.70	3.19	11.7	0.342
mEx.Me	-2.49	1.26	-2.21	0.990	12.9	0.215
mEx.Max	8.21	1.95	7.62	1.54	7.65	0.101
ACdAB.Covp	57.8	6.77	58.3	5.01	-0.88	0.668
OvD.Me	-16.8	2.08	-14.6	1.30	15.6	<0.001
OvD.Max	-10.1	1.99	-8.41	1.31	20.1	<0.001

Abbreviations are the same as in Table 1

**Figure 4:**
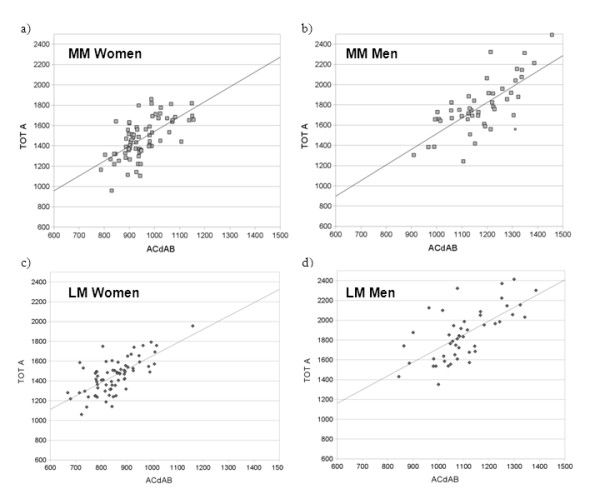
**Correlation of the total surface area (TOT A) of the meniscus with the ipsi-compartimental tibial plateau area (ACdAB); a) medial meniscus (MM) versus MT in women**. b) medial meniscus (MM) versus MT in men. c) lateral meniscus (LM) versus LT in women. d) lateral meniscus (LM) versus LT in men.

The TOT A of the menisci displayed a very high correlation with the meniscal volume medially (r = .97 for men and r = .96 for women) and laterally (r = .98 in men; r = .95 in women) and the meniscal width (r = .64 - .75; Tables 3, 4). TOT A also showed a moderate correlation with Th.Me (r = .50 - .74; Tables 3, 4), EA displaying higher correlations than FA and TA (data not shown). There was no significant correlation between measures of meniscus size and age in either men or women (data not shown). TOT A displayed significant correlations with the ipsi-compartimental ACdAB medially and laterally (r = 0.62 - 0.75; Figure 4), and these coefficients were greater than those between TOT A and contra-lateral or total ACdAB, or with body height or weight (Tables 3, 4).

**Table 3:** Correlation between measures of the medial meniscus size, tibial plateau area, and body measures in men/women

	TOT A	Th.Me	Wid.Me	Body Height	Body Weight	MT. ACdAB	**LT**. ACdAB	TOT. ACdAB
V	.**97**/.**96**	.**81**/.**82**	.**78**/.**76**	.27/.17	.18/-.05	.**64/.60**	.**55**/.13	**.65/.43**
TOT A		.**65/.66**	.**75**/.**68**	.27/.22	.16/-.03	.**72**/.**67**	.**58**/.23	**.72/.53**
Th.Me			**.59/.55**	.20/.07	.18/-.04	.35^x^/.34^x^	.36^x ^/.06	.39^x ^/.16
Wid.Me				.07/.07	.03/-.10	.35^x^/.37^x^	.41^x ^/.03	.42^x ^/.24
Body Height					.35^x ^/.**58**	**.61**/.**55**	**.53**/.**57**	**.63**/.**66**
Body Weight						.31/.19	.10/.32^x^	.22/.31^x ^
MT. ACdAB							.**66**/.**43**	.**91**/.**84**
LT. ACdAB								.**91**/.**85**

Abbreviations see above, please note that bold letters indicate p < 0.001, and (^x^) indicates p < 0.05

**Table 4:** Correlation between measures of the lateral meniscus size, tibial plateau area, and body measures in men/women

	TOT A	Th.Me	Wid.Me	Body Height	Body Weight	MT. ACdAB	LT. ACdAB	TOT. ACdAB
V	.**98**/.**95**	.**85**/.**73**	.**57**/.**61**	.17/.**51**	-.01/.24	.39^x ^/.**50**	.**64**/.**68**	.**56**/.**70**
TOT A		.**74**/.**50**	.**64**/.**64**	.19/.**53**	.02/.22	.37^x ^/.**48**	.**62**/.**75**	.**54**/.**73**
Th.Me			.29/.26^x^	.09/.27^x^	-.04/.21	.33/.33^x^	.**55**/.31^x^	.48^x ^/.38^x^
Wid.Me				.06/.21	.06/-.07	.06/.27^x^	.01/.14	.04/.24
Body Height					.35/.**58**	.**61**/.**55**	.**53**/.**57**	.**63**/.**66**
Body Weight						.31/.19	.10/.32^x^	.22/.31^x^
MT. ACdAB							.**66**/.**43**	.**91**/.**84**
LT. ACdAB								.**91**/.**85**

Abbreviations see above, please note that bold letters indicate p < 0.001 and (^x^) indicates p < 0.05

The ratio between TOT A and (the ipsi-compartimental) ACdAB was similar between men and women medially (1.52 ± 0.16 vs. 1.56 ± 0.14; p = 0.22; Table 1) and laterally (1.68 ± 0.20 vs. 1.70 ± 0.13; p = 0.72; Table 2; Figure 3). The within-sex, inter-subject variability for TOT A/ACdAB was considerably smaller than that for TOT A (CV% in men 11% vs. 16% medially/12% vs. 14% laterally, and in women 9% vs. 12% medially/8% vs. 12% laterally).

The relative position of the meniscus compared with the tibial plateau was similar between men and women in both medial menisci and lateral menisci when using percent coverage of the tibia by the meniscus (49.6 vs. 49.8% medially and 57.8 vs. 58.3% laterally; Table 1, 2). When using an absolute (size-dependent) measure, men displayed a significantly larger overlap distance between meniscus and tibia than women, both medially and laterally (Tables 1, 2). However, the meniscus surface not covering (i.e. extruding) the medial ACdAB (TA.uncovp) was significantly larger in women than in men (12.2% vs. 10.0%; Table 1), whereas laterally the values were not significantly different (p = 0.34; 6.4% in men vs. 5.7% in women; Table 2). Also, the mean "physiological" extrusion distance of medial menisci (but not that of lateral menisci) was significantly greater in women (1.83 ± 1.06mm) than in men (1.24 ± 1.18mm; p = 0.011; Table 1).

## Discussion

To our knowledge, no previous paper has examined a comprehensive set of quantitative measures of meniscus size and position between asymptomatic men and women without radiographic signs of knee OA. In this study we explored potential sex-differences in meniscus morphology, in view of women being more likely to develop knee OA than men. Also, we explored the correlation of meniscus morphology with body height, weight, and tibial plateau area, to propose a "relative measure" of meniscus size that can be directly compared between men and women, and that can be potentially used to efficiently explore whether the meniscus is hypertrophied or has lost substance in knee OA [17,19] in mixed cohorts of men and women. We find that men have significantly greater (absolute) tibial and meniscus surface areas than women as well as meniscus thickness and volume. Ipsi-compartimental tibial plateau area was more strongly correlated with medial (and lateral) meniscus size in men and women than body height or weight, and no significant correlation was observed with age. A relative measure of meniscus size (total meniscus surface area divided by ipsi-compartimental tibial surface area) was similar in men and women and was less variable between subjects (within each sex) than the non-normalized meniscal surface area. The coverage of the tibial plateau was similar between men and women, but the "physiological" medial meniscal extrusion was somewhat greater in women than in men.

A limitation of the study is that, although knees with MRI signs of meniscus lesions were excluded, the cartilage or ligament status of the knees, the limb alignment, and the radiographic biomechanical appearance of the pelvis and hip joint were not examined. Therefore, we cannot exclude with certainty, that some participants had early cartilage or ligamentous changes or deviations from neutral alignment. Further, we cannot exclude that some women had "male-like-shaped" pelvices and thus biomechanical conditions that were more similar to men than those of other women. However, all subjects were asymptomatic, radiographically normal, had no trauma history or any other risk factors of OA, and had no meniscus lesions. A further limitation of the study is the use of only coronal MR images, which are ideal for assessing the meniscal body (and external extrusion), but display partial volume effects in the anterior and posterior horns and thus cannot be used to evaluate anterior (or posterior) meniscus extrusion [35]. Meniscal surfaces and volumes might thus be somewhat larger than those reported here, but this effect is likely similar in men and women, so that the comparison between men and women is likely not substantially affected.

The meniscal volumes (and other measures) in our study are very similar to those given for women by Wirth et al. [17] using the same software, and were somewhat smaller than that given for a single (medial and lateral) cadaver meniscus (sex not reported) by Bowers et al. [36]. When comparing in situ MRI-based measurements (also using a T2*-weighted gradient echo sequence), these authors [36] reported a high degree of agreement with water-displacement of the surgically removed meniscus.

Our data extend previous findings [37-39] that meniscus length and width are larger in men than in women. The comprehensive set of morphometric parameters reported in our study may help in the design sex- and patient-specific meniscus transplants that are tailored to the size of the individual tibial plateau area. Stone et al. [38] reported a moderate to high correlation of both the tibial plateau width and meniscal length/width with body height, but a lower correlation of these with body weight in a mixed cohort of men and women. Our results confirm this relationship within asymptomatic men and women, and further show that the correlation between (ipsi-compartimental) tibial plateau size and meniscus size is greater than that with body height and weight. The total surface area of the meniscus (TOT A) showed very high correlations with meniscus volume and a moderate to high correlation with meniscus thickness and therefore provides a good and representative measure of meniscus size. When normalizing meniscus size (i.e. total surface area) to ipsi-compartimental tibial plateau size, the inter-subject variability within each sex was reduced. Further, sex-differences became minimal, women showing marginally greater (but not significantly greater) values than men. If meniscus size is to be compared between cohorts of OA and reference participants in context of studying meniscus hypertrophy or maceration in OA [17,19], the normalized meniscus surface area therefore provides an ideal parameter to explore this question in mixed cohorts of men and women, and to attain high statistical power.

A limitation of this cross-sectional study design is that the causality between meniscus morphology in asymptomatic knee without radiographic knee OA, and the development of knee OA cannot be established. Hence, the results can only provide hints as to why women may have a higher prevalence of knee OA than men [1]. Given that meniscal extrusion is known to contribute to the development (and progression) of knee OA [10-16], the finding of greater "physiological" medial meniscus extrusion in asymptomatic women compared with asymptomatic men is interesting. Whether this contributes to women having at greater risk of developing symptomatic knee OA than men [1-3] remains to be established in long-term follow-up studies in participants with incident knee OA. One needs to take into account that given subtle (albeit not statistically significant) size differences of the meniscus between men and women, the position of its internal margin and the percentage of tibial plateau coverage were very similar in both sexes. Therefore, unfavourable biomechanical protection of the tibial cartilage (by the meniscus) in women is unlikely, and tibial plateau coverage by the meniscus was similar between men and women, both medially and laterally. Nevertheless, greater "physiological" meniscus extrusion may potentially contribute to the incidence of knee symptoms.

## Conclusion

In conclusion, we found that medial and lateral tibial plateau and meniscal surface areas were larger in asymptomatic men than in women. Meniscus size correlated more strongly with the ipsi-compartimental tibial plateau size than with contra-compartimental or total tibial plateau size, body height and weight, and no significant correlation was observed with age. A relative measure of meniscus size (total meniscus surface area divided by ipsi-compartimental tibial surface area = TOTA/ACdAB) was less variable between subjects than non-normalized meniscal surface areas, and was not significantly different between both sexes. TOTA/ACdAB thus provides a useful measure to compare meniscus size across different cohorts containing men and women. Although tibial plateau coverage by the meniscus was similar in asymptomatic men and women, the "physiological" medial meniscal extrusion was greater in women than in men.

## List of abbreviations

3D: 3 dimensional; AC: area of cartilage; ACdAB: area of cartilage surface, including denuded areas of subchondral bone; ACdAB.Cov: area of cartilage surface covered with meniscus; ACdAB.Uncov: area of cartilage surface uncovered with meniscus; BMI: body mass index; Bul.Me: mean bulging of the meniscus; DESS: double echo steady state; Diff (%): difference in percent; EA: external area of the meniscus; Ex.Max: maximal extrusion; Ex.Me: mean extrusion; FA: femoral area of the meniscus; IW: intermediately weighted; LM: lateral meniscus; LT: lateral tibia; MM: medial meniscus; MM/LM.Ex.Me: mean extrusion of the medial/lateral meniscus; MM/LM.Th.Max: maximal thickness of the medial/lateral meniscus; MM/LM.Th.Me: mean thickness of the medial/lateral meniscus; MM/LM.V: volume of the medial/lateral meniscus; MM/LM.Wid.Me: mean width of the medial/lateral meniscus; MR: Magnetic resonance; MT: medial tibia; MT/LT.ACdAB.Cov: medial/lateral tibial plateau covered with meniscus; MT/LT.ACdAB.Uncov: medial/lateral tibial plateau uncovered with meniscus; OA: Osteoarthritis; OAI: Osteoarthritis Initiative; OARSI: Osteoarthritis Research Society International; OvD.Max: maximal overlap distance; OvD.Me: mean overlap distance; SD: standard deviation; TA: tibial area of the meniscus; TA.uncov: tibial area of the meniscus not covering the tibial plateau; Th.Max: maximal thickness of the meniscus; Th.Me: mean thickness of the meniscus; TOT A: total surface area of the meniscus; TSE: turbo spin echo; V: volume of the meniscus; Wid.Max: maximal width of the meniscus; Wid.Me: mean width of the meniscus.

## Conflict of interest statement

Felix Eckstein is CEO and co-owner of Chondrometrics GmbH, a company providing MR image analysis services. He provides consulting services to MerckSerono, Novartis and Sanofi Aventis. Wolfgang Wirth also and Martin Hudelmaier are partially employed by Chondrometrics GmbH, and Wolfgang Wirth is a co-owner of Chondrometrics GmbH. Rainer Burgkart as the head of research of the Clinic for Orthopaedics and Traumatology of the Technische Universität München, Martin Englund, Richard Frobell and Katja Blöcker have no conflict of interest.

## Authors' contributions

KB., MH and FE have made substantial contributions to the study conception and design, the analysis and interpretation of data, drafting the article and final approval of the version of the article to be published. RF and ME conceived the idea of studies of normal meniscus MRI morphology quantitatively and semi-quantitatively in the OAI "non-exposed" cohort. In addition, WW, ME, RB and RF have made substantial contributions to the analysis and interpretation of data, to revising the article critically for important intellectual content, and to final approval of the version of the article to be published.

## Pre-publication history

The pre-publication history for this paper can be accessed here:

http://www.biomedcentral.com/1471-2474/12/248/prepub
